# *Origanum majorana* L.: A Nutritional Supplement With Immunomodulatory Effects

**DOI:** 10.3389/fnut.2021.748031

**Published:** 2021-09-22

**Authors:** Senye Wang, Li Zhou, Fatma Al-Zahra K. K. Attia, Qi Tang, Mengke Wang, Zhenhua Liu, Geoffrey I. N. Waterhouse, Lijun Liu, Wenyi Kang

**Affiliations:** ^1^National R&D Center for Edible Fungus Processing Technology, Henan University, Kaifeng, China; ^2^Joint International Research Laboratory of Food and Medicine Resource Function, Kaifeng, China; ^3^Department of Ornamental, Medicinal and Aromatic Plants, Faculty of Agriculture, Assiut University, Asyut, Egypt; ^4^School of Chemical Sciences, University of Auckland, Auckland, New Zealand; ^5^Huaihe Hospital, Henan University, Kaifeng, China

**Keywords:** *Origanum majorana* L., procumboside B, immune, NF-κB, MAPKs, RAW264.7

## Abstract

*Origanum majorana* L. is an aromatic herb that has been grown in several Mediterranean countries since ancient times, but became popular during the Middle Ages as a medicinal plant and seasoning ingredient. *O. majorana* has many pharmacological effects, but its immunoreactive components and mechanisms are still unclear. In this study, four compounds were isolated and identified from *O. majorana* by a spectral analysis, including ^1^H and ^13^C-NMR. They were 1H-indole-2-carboxylic acid (**1**), (+)-laricresol (**2**), (+)-isolaricresol (**3**), and procumboside B (**4**, pB), which were isolated for the first time in *O. majorana*. The immunomodulatory effects of the four compounds were screened, and pB had good immunomodulatory activity on RAW 264.7 cells. The immunomodulatory mechanism of pB was proved, in which pB could increase the secretion of nitric oxide (NO), interleukin-6 (IL-6), tumor necrosis factor-α (TNF-α), and reactive oxygen species (ROS) and simultaneously upregulate the expression of CD80 and CD86 on the cell surface. These results suggested that the mechanism of pB may be related to the activation of nuclear factor-kappaB (NF-κB) and mitogen-activated protein kinases (MAPKs)-signaling pathways. *O. majorana* is rich in nutrients and is commonly used in diets, so it can be used as a nutritional supplement with immunomodulatory effects.

## Introduction

*Origanum majorana* L. is a perennial herb of the Lamiaceae family that is mainly distributed in the Mediterranean region, especially in Morocco, Algeria, and Egypt (1). The chemical constituents in *O. majorana* are mainly essential oils and other components, such as polyphenols, flavonoids, sterols, triterpenes, alkaloids, coumarins, tannins, and saponins (2). The essential oils consist of terpineol (29.6%), 2-carene (20.1%), camphene (13.4%) and α-pinene (7.9%), which have antibacterial effects (2). In addition, phenolic acids are also the main components in *O. majorana*, including gallic acid, caffeic acid, dihydroxy acid, and chlorogenic acid (3, 4).

*Origanum majorana* is an important aromatic plant whose essential oils and polyphenols from its leaves, stems, and flowers are commonly used in cookery as a spice and condiment (5). Polyphenols have strong antioxidant effects, often used as food additives (6). *O. majorana* can be made into a tea and used to protect the hormone levels of women (7). Also, the plants contain carbohydrates, proteins, amino acids, and vitamin C and are widely used in food industries (8, 9).

In addition to the direction of food industries, *O. majorana* has many medicinal functions. Methanol extracts and essential oils of this plant have bacteriostatic, anticancer, antioxidant, and insecticidal effects (10, 11). The phenolic compounds of *O. majorana* have antidiabetic properties involving increased plasma insulin, the stimulation of liver glycogen synthesis, and increased glucokinase activity. In addition, *O. majorana* was reported to have nephrotoxicity protective, anti-inflammatory, analgesic, and antipyretic effects (6, 12). However, its immune effect has not been reported yet, and its active components and mechanism are still unclear. Therefore, it is necessary to conduct immunomodulatory studies on the chemical constituents of *O. majorana*.

Macrophages, as an important part of innate immunity, play an irreplaceable role in immune regulation (13). In macrophages, mitogen-activated protein kinases (MAPKs) and nuclear factor-kappaB (NF-κB) are the main immunomodulatory-signaling pathways reported (14, 15). The activation of NF-κB and MAPKs-signaling pathways induces the expression of inflammatory genes and increases the secretion of immune molecules, including nitric oxide (NO), TNF-α, interleukin-1 (IL-1), IL-6, and IL-12, leading to a systemic inflammatory response and the clearance of pathogens (16, 17). Reactive oxygen species (ROS) are also produced by activated macrophages as immunomodulatory-signaling molecules in macrophages, like other pro-inflammatory cytokines (18, 19). Macrophages are involved in triggering the primary responses of the adaptive immune system by processing and presenting antigens (20). In response to immune challenges, macrophages are activated to increase the expression of the major histocompatibility complex (MHC) and differentiated molecule (CD) on the cell surface to process presenting antigens and, thus, effectively remove pathogens (21, 22). Macrophages RAW264.7 could more realistically simulate the immune response of human macrophages cultured *in vitro*. At the same time, mouse macrophages RAW264.7 is a representative cell line for the *in vitro* study of immune activity in many kinds of literature (23, 24). Therefore, RAW264.7 macrophages were selected as the experimental model of this study.

**Figure 1:**
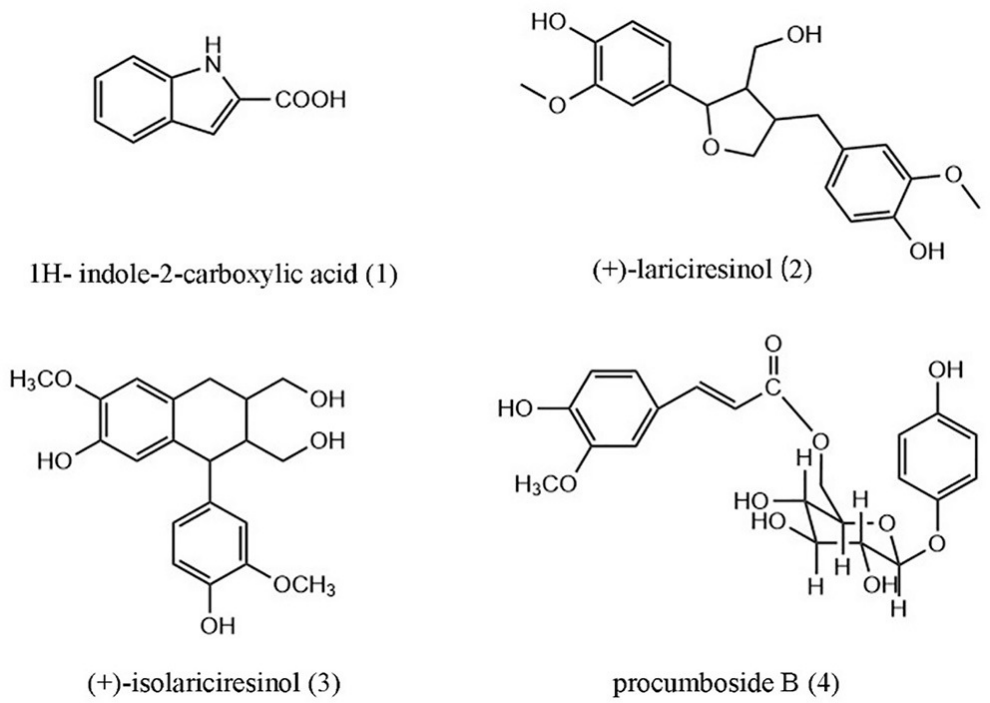
Structures of compounds **1**–**4** from *Origanum majorana*.

In this study, four compounds were isolated from the 40% ethanol extract of *O. majorana*, including 1H-indole-2-carboxylic acid (**1**), (+)-lariciresinol (**2**), (+)-isolariciresinol (**3**), and procumboside B (**4**, pB) (Figure 1). The immune effects of the compounds were screened, and the results showed that pB had good effect on RAW264.7 cells. Its mechanism was elucidated by Western blot experiments and reverse transcription-PCR (RT-PCR) experiments. The results prove that *O. majorana* can be used as nutritional supplements.

## Materials and Methods

### Reagents and Instruments

The following technologies were used in this research: NMR spectra were performed on Bruker AM-400 instruments with TMS as the internal standard (Bruker, Bremerhaven, Germany); column chromatography was performed by silica gel (200–300 and 300–400 mesh, Qingdao Marine Chemical Inc., Qingdao, China); Rp-18 gel (40–63 μm, Merck, Darmstadt, Germany); Sephadex LH-20 (20–150 μm, Amersham Biosciences, Uppsala, Sweden); automatic microplate reader (Thermo Fisher Scientific, Shanghai, China); QuanStudio 3 Real-Time PCR (Thermo); NanoDrop2000c Spectrophotometer (Thermo); Fluor Chem M (Protein simple); flow cytometer (BD Facsverse, BD Biosciences, Franklin Lakes, NJ, USA).

The following reagents were used in this research: RAW264.7 cells were purchased from the Typical Culture Preservation Committee Cell Bank, Chinese Academy of Sciences; Dulbecco's modified Eagle's medium (DMEM) (ProCell, Wuhan, China); a penicillin-streptomycin mixture, nucleoprotein extraction kit, BCA protein quantitative kit, and protease phosphatase inhibitor (SolarBio, Beijing, China); fetal bovine serum (FBS) (Gibco, Grand Island, NE USA); LPS (Sigma-Aldrich, St. Louis, MO, USA); NO kit and ROS kit (Beyotime Biotechnology, Shanghai, China); mouse TNF-α ELISA kit and mouse IL-6 ELISA kit (4A Biotech Co, Ltd., Beijing, China); *Evo M-MLV* RT Kit with gDNA Clean and SYBG Green Premix *Pro Taq* HS qPCR kit (Accurate Biotechnology, Hunan, China); antibody NF-κBP65, p-NF-κBP65, P38, p-P38, SAPK/JNK, p-SAPK/JNK, P44/P22 ERK, and p-P44/P22 ERK (Cell Signaling Technology, Danvers, MA, USA); primers were designed and synthesized by Thermo Fisher Scientific (Shanghai, China). Primer sequences are shown in Table 1.

**Table 1:** Primers for reverse transcription-PCR (RT-PCR).

**Gene**	**Sequences**
iNOS	Forward: 5′ - GCTCGCTTTGCCACGGACGA -3′ Reverse: 5′ - AAGGCAGCGGGCACATGCAA -3′
TNF-α	Forward: 5′ - CCCTCCTGGCCAACGGCATG -3′ Reverse: 5′ - TCGGGGCAGCCTTGTCCCTT -3′
IL-6	Forward: 5′ - AGACAAAGCCAGAGTCCTTCAGAGA -3′ Reverse: 5′ - GCCACTCCTTCTGTGACTCCAGC -3′
β-actin	Forward:5′- TGCTGTCCCTGTATGCCTCT-3′ Reverse:5′- TTTGATGTCACGCACGAT TT-3′

### Extraction and Isolation

The dried aerial parts of *O. majorana* (4.9 kg) were extracted with a petroleum ether at room temperature for 72 h to remove the volatile oil. The filter residue was soaked and extracted three times (each time for 7 days) with 70% ethanol at room temperature and then filtered and evaporated under reduced pressure to obtain a crude extract (1,360 g).

The crude extract was chromatographed on D101 macroporous resin and eluted with water, 20% ethanol, 40% ethanol, 60% ethanol, and 95% ethanol, respectively. Then, five fractions were obtained (Fr. A–Fr. E). The 40% ethanol fraction, Fr. B (115 g), was subjected to silica gel column chromatography, eluting with a gradient of CHCl_3_-MeOH (40:1~1:1) to obtain eight fractions (Fr. B-1~Fr. B-8) by a TLC plate analysis.

Fr. B-2 (2.01 g) was subjected to silica gel column chromatography, eluted with CHCl_3_-EtOAc (6:1, v/v), to obtain four fractions (Fraction 1 to Fraction 4). Fraction 2 (725 mg) was separated by Sephadex LH-20 column chromatography (MeOH) and then purified by semi-preparative HPLC (MeOH-H_2_O, 57:43, v/v) to obtain compound **1** (3.4 mg) and compound **2** (9.5 mg).

Fr. B-3 (900 mg) was separated by Sephadex LH-20 column chromatography (MeOH) to obtain six fractions (Fraction 1 to Fraction 6). Fraction 2 (385 mg) was subjected to silica gel column chromatography, eluted with CHCl_3_-MeOH (6:1, v/v), and then purified by semi-preparative HPLC (MeOH-H_2_O, 65:35, v/v) to obtain compound **3** (33 mg).

Fr. B-4 (3.1 g) was separated by an automatic preparation of liquid chromatography (MeOH-H_2_O, 55:45, v/v) to obtain four fractions (Fraction 1 to Fraction 4). Fraction 1 (1.04 g) was subjected to silica gel column chromatography, eluted with a system of EtOAc-MeOH (10:1, v/v), and then purified by Sephadex LH-20 (MeOH) to obtain compound **4** (237 mg).

Compound **1**: 1H-indole-2-carboxylic acid, pale yellow crystal; ^1^H-NMR (CD_3_OD, 400 MHz) δ: 7.95 (1H, s, H-3), 8.08 (1H, dd, *J* = 6.4, 2.3 Hz, H-4), 7.21~7.14 (2H, m, H-5, 6), 7.43 (1H, m, H-7). ^13^C-NMR (CD_3_OD, 100 MHz) δ: 133.3 (C-2), 108.8 (C-3), 122. (C-4), 123.6 (C-5), 122.3 (C-6), 112.9 (C-7), 138.2 (C-8), and 127.6 (C-9). Its data were in good accordance with those of 1H- indole-2-carboxylic acid (25).

Compound **2**: (+)-lariciresinol, pale yellow jelly; ^1^H-NMR (CD_3_OD, 400 MHz) δ: 6. 91 (1H, d, H-2), 6.77 (2H, d, H-5, H-6), 4.75 (1H, d, *J* = 6.9 Hz, H-7), 2.38 (1H, m, H-8), 3.63 (1H, dd, *J* = 11, 6.5 Hz, Ha-9), 3.81 (1H, overlapped, Hb-9), 6.80 (1H, d, *J* = 1.9 Hz, H-2′), 6.70 (1H, m, H-5′), 6.64 (1H, dd, *J* = 8., 1.9 Hz, H-6′), 2.49 (1H, dd, *J* = 13.4, 11.3 Hz, Ha-7′), 2.93 (1H, dd, *J* = 13.4, 4.8 Hz, Hb-7′), 2.73 (1H, m, H-8′), 3.72 (1H, dd, *J* = 8.4, 5.8 Hz, Ha-9′), 3. 98 (1H, dd, *J* = 8.4, 6.4 Hz, Hb-9′), 3.83 (3H, s, 3-OMe), 3.84 (3H, s, 3′-OMe); ^13^C-NMR (CD_3_OD, 100 MHz) δ: 135.7 (C-1), 110.6 (C-2), 149. (C-3), 147. (C-4), 116. (C-5), 119.8 (C-6), 84. (C-7), 54.1 (C-8), 60.4 (C-9), 133.5 (C-1′), 113.4 (C-2′), 149. (C-3, C-3′), 145.8 (C-4′), 116.2 (C-5′), 122.2 (C-6′), 33.7 (C-7′), 43.9 (C-8′), 73.5 (C-9′), 56.3 (3-OMe), and 56.3 (3′-OMe). Its data were in good accordance with those of (+)-lariciresinol (26).

Compound **3**: (+)-isolariciresinol, pale yellow jelly; ^1^H-NMR (CD_3_OD, 400MHz) δ: 6. 68 (1H, d, *J* = 1.5 Hz, H-2), 6.74 (1H, d, *J* = 8. Hz, H-5), 6.61 (1H, dd, *J* = 8., 1.7Hz, H-6), 3.82 (1H, overlapped, H-7), 1.77 (1H, m, H-8), 3.68 (3H, m, Ha-9, H-9′), 3.40 (1H, dd, *J* = 11.2, 4. Hz, Hb-9), 6.65 (1H, s, H-2′), 6.18 (1H, s, H-5′), 2.78 (2H, d, *J* = 7.7 Hz, H-7′), 2.00 (1H, m, H-8′), 3.80 (3H, s, 3′-OMe), 3.79 (3H, s, 3-OMe); ^13^C-NMR (CD_3_OD, 100 MHz) δ: 138.6 (C-1), 113.7 (C-2), 149. (C-3), 145.9 (C-4), 116. (C-5), 123.2 (C-6), 48. (C-7), 48. (C-8), 62.2 (C-9), 129., (C-1′), 112.3 (C-2′), 147.1 (C-3′), 145.2 (C-4′), 117.3 (C-5′), 134.1 (C-6′), 33.6 (C-7′), 39.9 (C-8′), 65.9 (C-9′), 56.3 (3-OMe), and 56.4 (3′-OMe). Its data were in good accordance with those of (+)-isolariciresinol (26).

Compound **4**: procumboside B, amorphous white powder; ^1^H-NMR (CD_3_OD, 400 MHz) δ: 696. (1H, d, H-2), 6.94 (1H, d, H-3), 6.66 (1H, d, H-5), 6.64 (1H, d, H-6), 4.73 (1H, d, *J* = 7.3 Hz, H-1′), 3.49~3.39 (3H, m, H-2′, 3′,4′), 3.65 (1H, m, H-5′), 4.54 (1H, dd, *J* = 11.6, 2.1 Hz, H-6a), 4.34 (1H, dd, *J* = 11.6, 5.6 Hz, H-6b), 7.18 (1H, d, H-2″), 6.83 (1H, d, H-5″), 7.08 (1H, dd, H-6″), 7.64 (1H, d, *J* = 15.9 Hz, H-7″), 6.39 (1H, d, *J* = 15.9 Hz, H-8″), 3.90 (3H, s, 3″-OCH_3_) 0.1^3^C-NMR (CD_3_OD, 100 MHz) δ: 153.9 (C-1), 119.5 (C-2), 116.6 (C-3), 152.3 (C-4), 116.6 (C-5), 119.6 (C-6), 103.7 (C-1′), 74.9 (C-2′), 77.9 (C-3′), 71.8 (C-4′), 75.4 (C-5′), 64.7 (C-6′), 127.7 (C-1″), 111.6 (C-2″), 150.7 (C-3″), 149.4 (C-4″), 116.5 (C-5″), 124.2 (C-6″), 147.1 (C-7″), 115.2 (C-8″), 119. (C-9″), 56.4 (3″-OCH_3_). Its data were in good accordance with those of procumboside B (27).

## Cell Culture and Treatment

### Cell Viability Assay

RAW264.7 cells were cultured by the assay that was the same as that of Junya Wang (28). RAW264.7 cells with logarithmic growth were inoculated in 96-well plates at a density of 1 × 10^4^ cells per well and incubated at 37°C and 5% CO_2_. When the cell fusion reached about 70–80%, the cell culture medium was removed, and the cells were treated with 100-μL DMEM, containing different concentrations of pB (6.25, 12.5, 25, 50, 100, and 200 μM) for 24 h. The blank group was given the same amount of complete DMEM without pB. Approximately 10-μL of MTT (0.5 mg/mL) were added to each well, followed by further incubation for 4 h at 37°C. Then, the cell culture medium was discarded and added to 100-μL of DMSO. The absorbance was measured at 490 nm to calculate the survival rate of cells.

### Nitric Oxide Assay

RAW264.7 cells with logarithmic growth were inoculated in 24-well plates at a density of 1 × 10^4^ cells per well and incubated with 500-μL DMEM at 37°C and 5% CO_2_. When the cell fusion reached about 70–80%, the cell culture medium was discarded. The LPS positive group was stimulated with 1-μg/mL LPS, and the pB group was stimulated with pB. After further incubation for 24 h, the cell culture medium was collected, and the levels of NO were determined according to the instructions of the manufacturer.

### Measurement of Cytokines by ELISA

Cell treatment was the same as above. The cell supernatants were collected, the dilution ratio was adjusted, and 100 μL of the sample was absorbed for content determination. The levels of TNF-α and IL-6 in RAW264.7 cells were measured by ELISA assay kits according to the instructions of the manufacturer.

### ROS Determination by Flow Cytometry

Cell treatment was the same as above. The content of ROS was determined by flow cytometry according to the experimental method in the literature of Honglin Wang (15). The cell culture medium was discarded, and 1-mL DCFH-DA (10 μM) was added to each well. Then, cells were incubated for 30 min at 37°C in the dark and washed three times with PBS to completely remove the DCFH-DA from the cells. The intracellular ROS levels were detected by detecting the changes in fluorescence intensity caused by the oxidation of the fluorescent probe DCFH-DA.

### CD80, CD86, and MHC II Determination by Flow Cytometry

Cell treatment was the same as above. The cells were collected and the cell density was adjusted to 1 × 10^6^ cells per 100 μL, then a 0.125-μg/100 mL antibody was added, and the cells were incubated for 30 min at 37°C in dark. After washing the cells with PBS three times, the expression of CD80, CD86, and MHC II on the cell surface was detected by flow cytometry.

### Western Blot

Cell treatment was the same as above. Cells were collected; total protein was extracted with the appropriate volume of a weak radio-immune precipitation assay (RIPA) lysis buffer depending on the density of the cultured cells (about 120 μL per well); and protein concentration was determined by the BCA method. Proteins (30 μg) were isolated by 10% sodium dodecyl sulfate–polyacrylamide gel electrophoresis (SDS-PAGE) and then transferred to polyvinylidene fluoride (PVDF) membranes. The membranes were blocked with 5% skim milk powder and hybridized with phosphorylated and non-phosphorylated antibodies overnight at 4°C. After being washed three times with Tris-buffered saline-Tween 20 (0.1%), the membranes were incubated with respective secondary antibodies conjugated with horseradish peroxidase for 2 h. Blots were visualized using enhanced chemiluminescence (ECL) detection kits and analyzed using the Image J software.

### Reverse Transcription-PCR (RT-PCR)

Cell treatment was the same as above. RAW264.7 cells were treated with LPS or pB for 12 h, and the cells were washed three times by PBS. Total RNA was isolated using Trizol reagent and reversed to cDNA by using *Evo M-MLV* RT Kit with gDNA Clean according to the instructions of the manufacturer. The RNA concentration and A260/A280 value of each sample were measured by NanoDrop2000 spectrophotometer (Thermo). Then, the expression of mRNAs was determined by the SYBR Green *Pro Taq* HS Premix qPCR kit. According to the ratio of the Ct value of the target gene to the Ct value of reference gene β-actin, the mRNA-relative expression level of the target gene in the samples was calculated by the 2^−Δ*ΔCt*^ method.

### Statistical Analysis

The experiments were repeated independently at least three times and analyzed statistically by SPSS 20 software. Data were expressed as mean ± SD. Statistical significance was calculated by a one-way ANOVA analysis.

## Results

### Effects of pB on RAW264.7 Cells Viability

**Figure 2:**
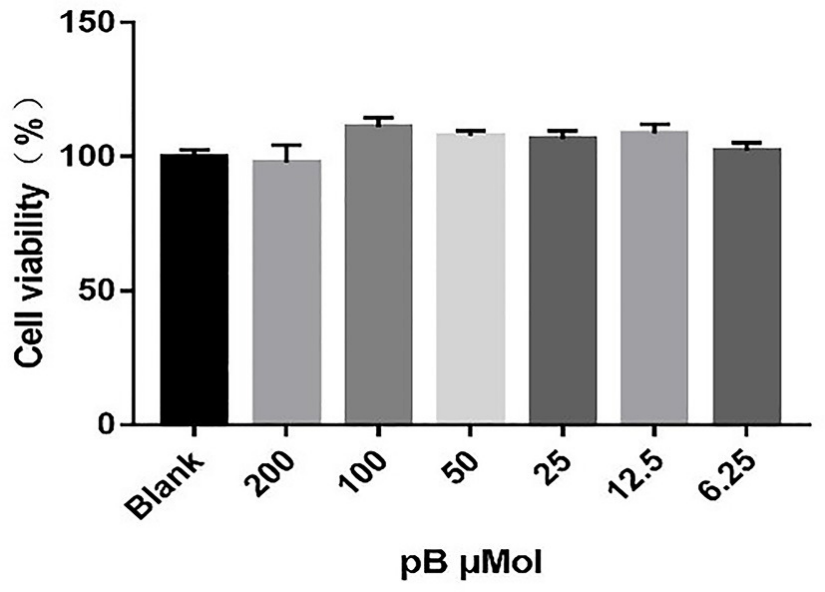
Effects of procumboside B (pB) on the viability of RAW264.7 cells. Data presented are the mean ± SD, *n* = 6.

To determine the non-toxic concentration range of pB, the effects of six concentrations of pB (200, 100, 50, 25, 12.5, and 6.25 μM) on the viability of RAW 264.7 cells were detected by an MTT assay. In Figure 2, compared with the blank group, pB had no significant effects on cell viability in the concentration range of 200–6.25 μM. Therefore, 100, 50, and 25 μM were selected as the study doses for this study.

### Effects of pB on NO Production and iNOS Expression

**Figure 3:**
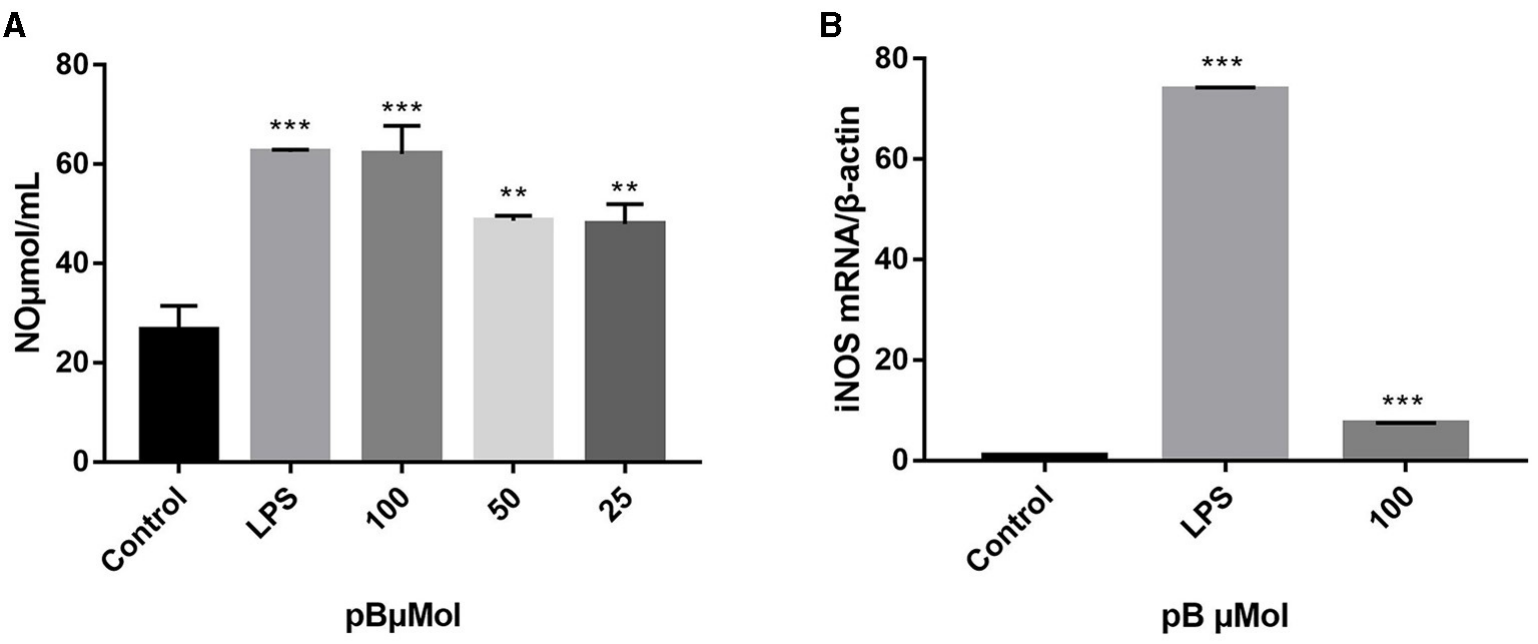
Effects of pB on nitric oxide (NO) production **(A)** and nitric oxide synthase (iNOS) mRNA expression **(B)** in RAW264.7 cells. Data presented are the mean ± SD, *n* = 3. Compared with the control, ^***^p < 0.001, ^**^p < 0.01.

Macrophage-derived NO plays an important role in immunity and maintaining homeostasis. Nitric oxide synthase (iNOS), as a pivotal enzyme in the inflammatory response of macrophages, has catalytic effects on the production of NO (29, 30). Therefore, we evaluated the immune efficacy of pB by detecting NO content and the relative mRNA expression level of iNOS in different groups. In Figure 3, the content of NO secreted in the control group was low, while the content of NO was significantly increased after LPS stimulation (*p* < 0.001). After pB stimulation, the release of NO was significantly higher than that of the control group (*p* < 0.001) with a dose-dependent manner, indicating that pB could activate NO release. The increased mRNA expression level of iNOS detected by RT-PCR further verified these results (Figure 3B). Thus, it was found that LPS and pB (100 μM) increased the mRNA expression levels of iNOS at 12 h of treatment (*p* < 0.001).

### Effects of pB on the Expression of TNF-α, IL-6, and mRNA in RAW264.7 Cells

**Figure 4:**
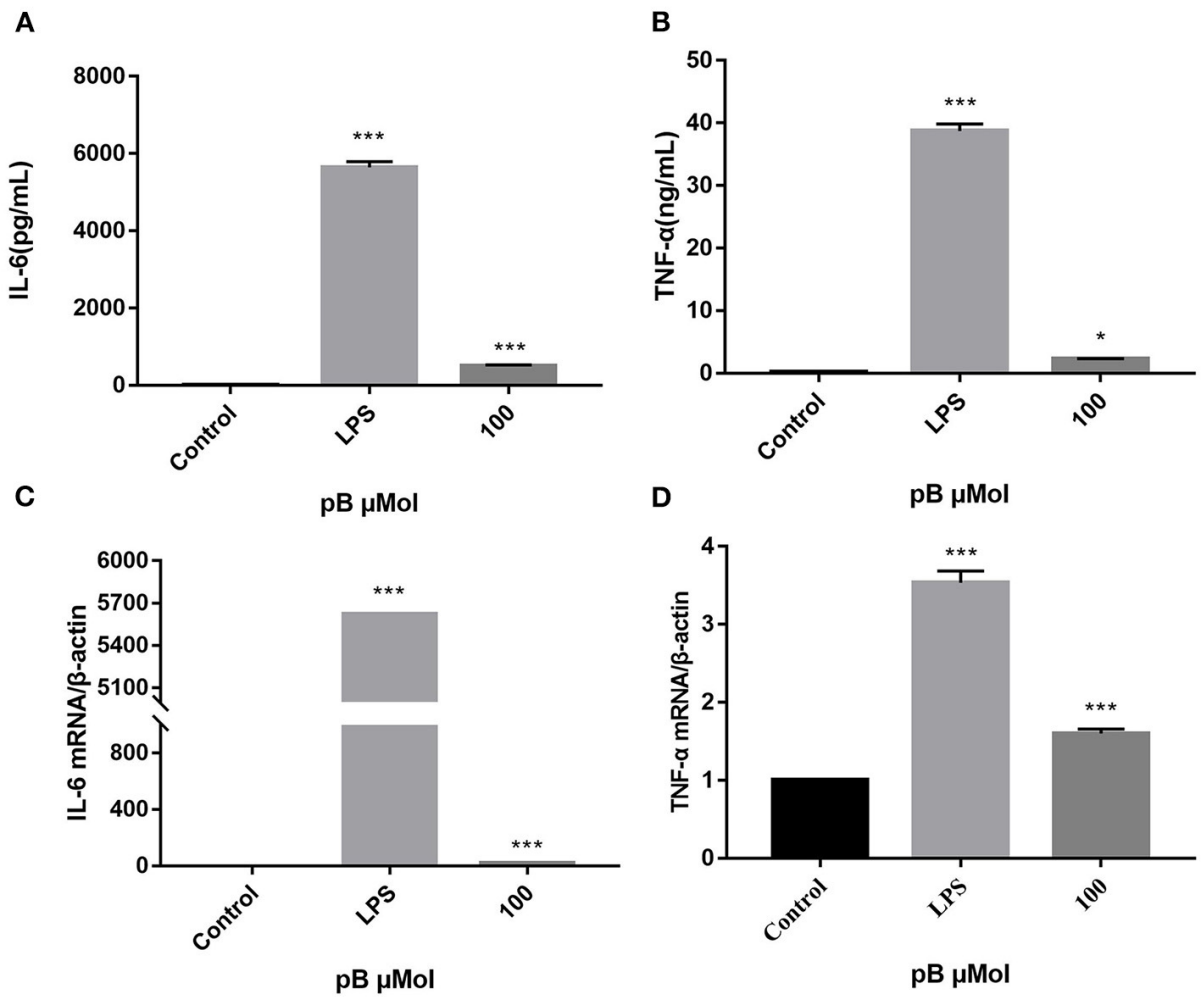
The effects of pB on the expression of IL-6 **(A)**, tumor necrosis factor-α (TNF-α) **(B)**, interleukin-6 (IL-6) **(C)**, and TNF-α **(D)** mRNA in Raw264.7 cells. Data presented are the mean ± SD, *n* = 3. Compared with the control, ^***^p < 0.001 and ^*^p < 0.05.

When macrophages are activated, more cytokines will be secreted, including TNF-α and IL-6, which all exert immunomodulatory effect. TNF-α can increase the production of a series of inflammatory cytokines and enhance the immune response of monocytes and macrophages. IL-6 is a pleiotropic pro-inflammatory cytokine whose release is associated with chronic inflammation and multiple factors of autoimmune disorders, which can promote the proliferation and differentiation of B cells and T cells (31–33). Therefore, the contents of TNF-α and IL-6 in the supernatant of different groups of cells were detected by ELISA. In Figures 4A–D, the levels of TNF-α and IL-6 and the expression of their corresponding mRNAs were significantly increased after LPS stimulation (1 μg/mL). After pB (100 μM) stimulation, the contents of TNF-α and IL-6 and the expression of the corresponding mRNA were also significantly increased compared with the control group (*p* < 0.001). These results suggested that pB could induce the corresponding mRNA expression and thus increase the secretion of cytokines.

### Effects of pB on the Activation of MAPKs and NF-κB Pathways in RAW264.7 Cells

**Figure 5:**
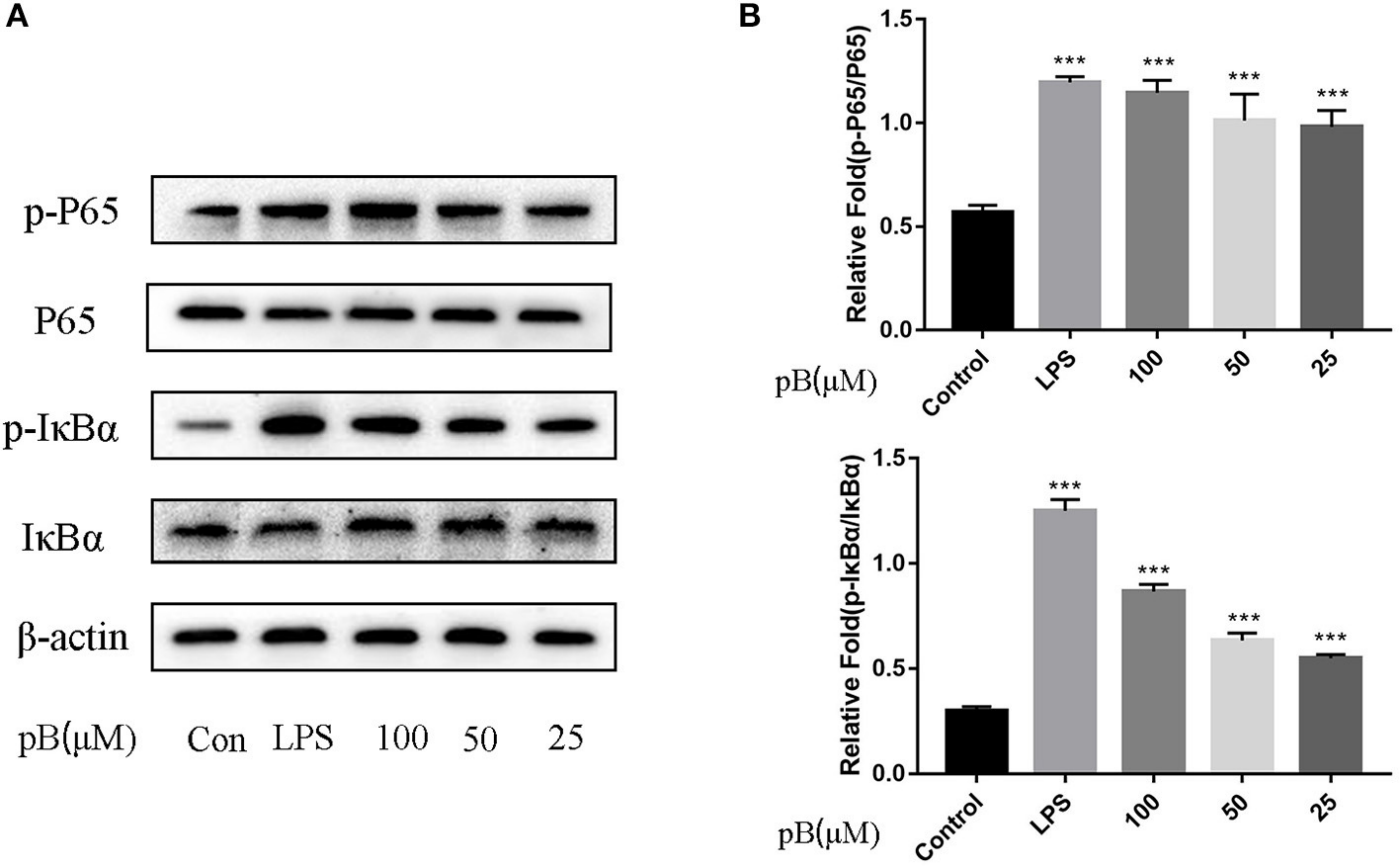
Effects of pB on the nuclear factor-kappaB (NF-kB) pathway with Western blotting. **(A)** The expression of p-P65, P65, p-IκBα and IκBα were detected using Western blotting. **(B)** The ratio of the content of p-P65/P65 and p-IκBα/IκBα. Data presented are the mean ± SD, *n* = 3. Compared with the control, ^***^p < 0.001.

**Figure 6:**
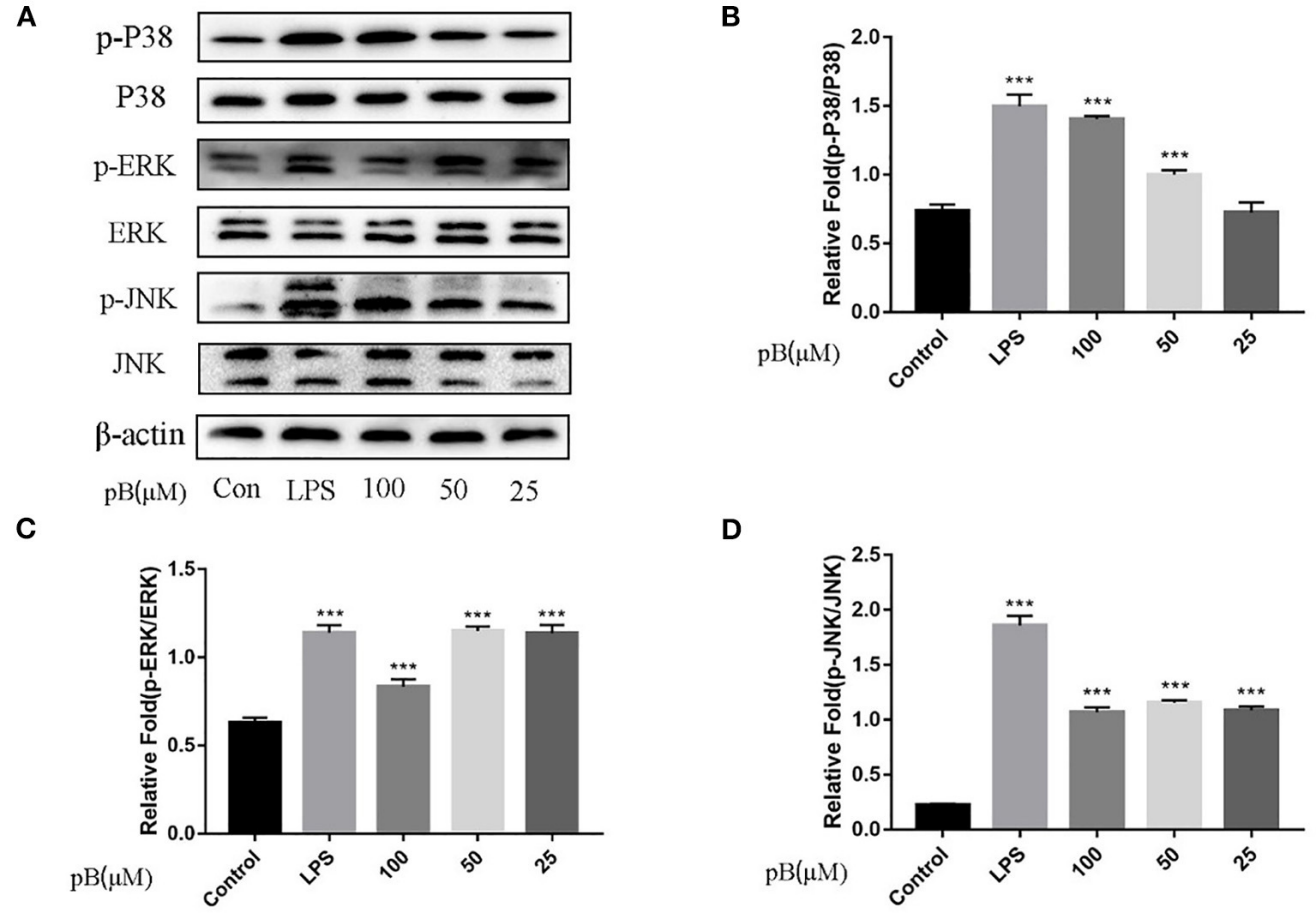
Effects of pB on the mitogen-activated protein kinases (MAPKs) pathway with Western blotting. **(A)** The expression of p-P38, P38, p-ERK, ERK, p-JNK, and JNK detected using Western blotting. **(B)** The ratio of the content of p-P38/P38. **(C)** The ratio of the content of p-ERK/ERK. **(D)** The ratio of the content of p-JNK/JNK. Data presented are the mean ± SD, *n* = 3. Compared with the control, ^***^p < 0.001.

In macrophages, NF-κB and MAPKs-signaling pathways are the main signaling pathways that control the immune response. The key proteins in the signaling pathway, as markers of signaling pathway activation, were selected, such as IκBα and P65 and their corresponding phosphorylation forms in the NF-κB-signaling pathway, extracellular signal–related kinase (ERK)-1/2, P38, c-Jun NH2-terminal kinase (JNK), and corresponding phosphorylation forms in the MAPKs-signaling pathway (15). In Figure 5, compared with the control group, the expression levels of phosphorylated P65 and phosphorylated IκBα were significantly increased in the LPS positive group. By calculation, the ratios of p-P65/P65 and p-IκBα/IκBα in the LPS positive group were significantly higher than those of the control group (*p* < 0.001). After pB stimulation, the ratios of p-P65/P65 and p-IκB-α/IκBα were also significantly higher than those of the control group. The results indicated that pB could induce immune response through the activation of the NF-κB-signaling pathway. In Figure 6, the ratios of p-P38/P38, p-ERK, and p-JNK in the LPS positive group were significantly higher than those in the control group (*p* < 0.001). After pB stimulation, the ratios of p-P38/P38, p-ERK/ERK, and p-JNK/JNK were significantly higher than those in the control group. The results indicated that pB could activate the MAPKs-signaling pathway to induce an immune response.

### Effects of pB on the Production of ROS in RAW264.7 Cells

**Figure 7:**
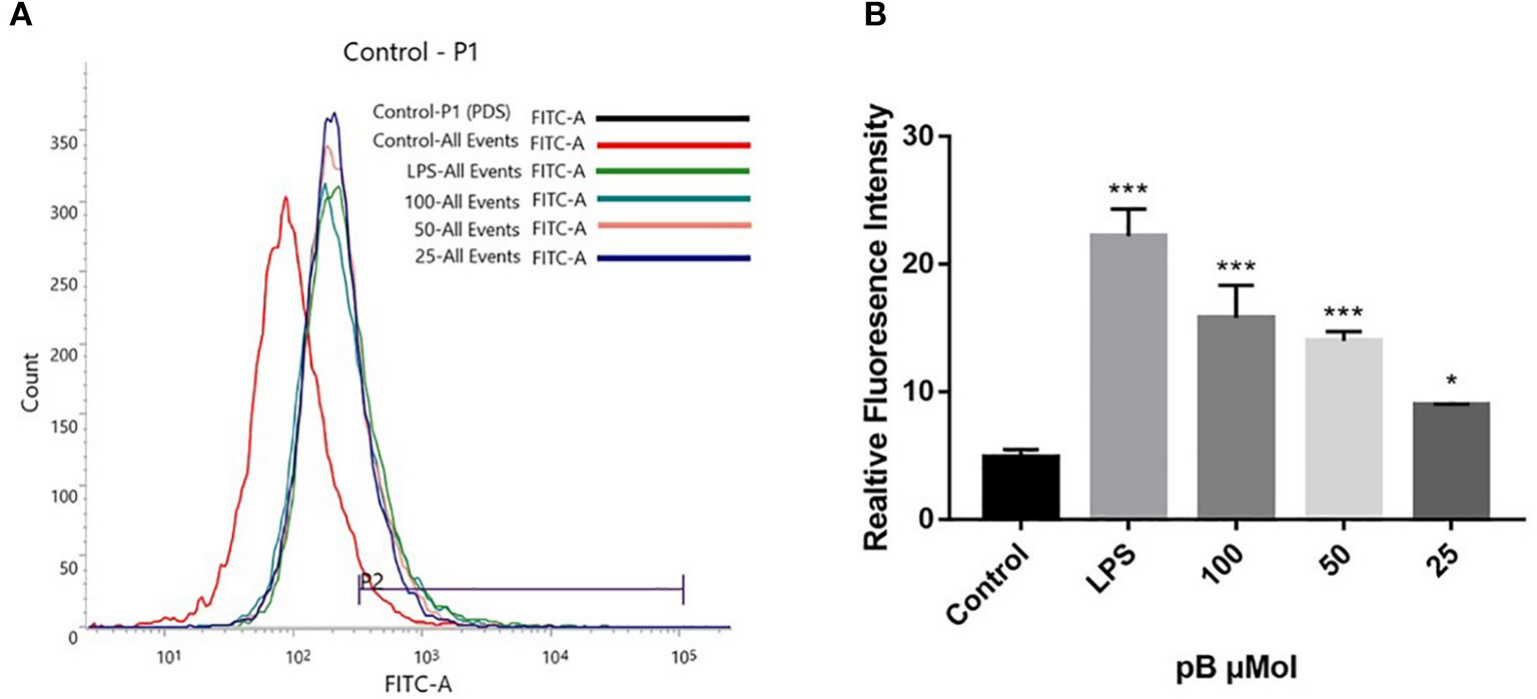
The effects of pB on reactive oxygen species (ROS) in RAW264.7 cells. **(A)** The fluorescence intensity was determined by flow cytometry at the FITC channel. **(B)** The realitive fluoresence intensity of ROS per group. Data presented are the mean ± SD, *n* = 3. Compared with the control, ^***^p < 0.001 and ^*^p < 0.05.

Reactive oxygen species act as immunomodulatory-signaling molecules in macrophages, as other pro-inflammatory cytokines do (34). Therefore, we used flow cytometry to detect the content of ROS. In Figure 7, compared with the control group, the peak shape of the LPS positive group moved to the right, which indicated that the ROS content in RAW264.7 cells was significantly increased after LPS stimulation. After pB stimulation, the peak shape also shifted to the right compared with the control group, indicating that ROS production increased significantly. This suggested that pB could further affect the immune response by producing ROS.

### Effects of pB on the Expression of CD86, CD80, and MHC II in RAW264.7 Cells

**Figure 8:**
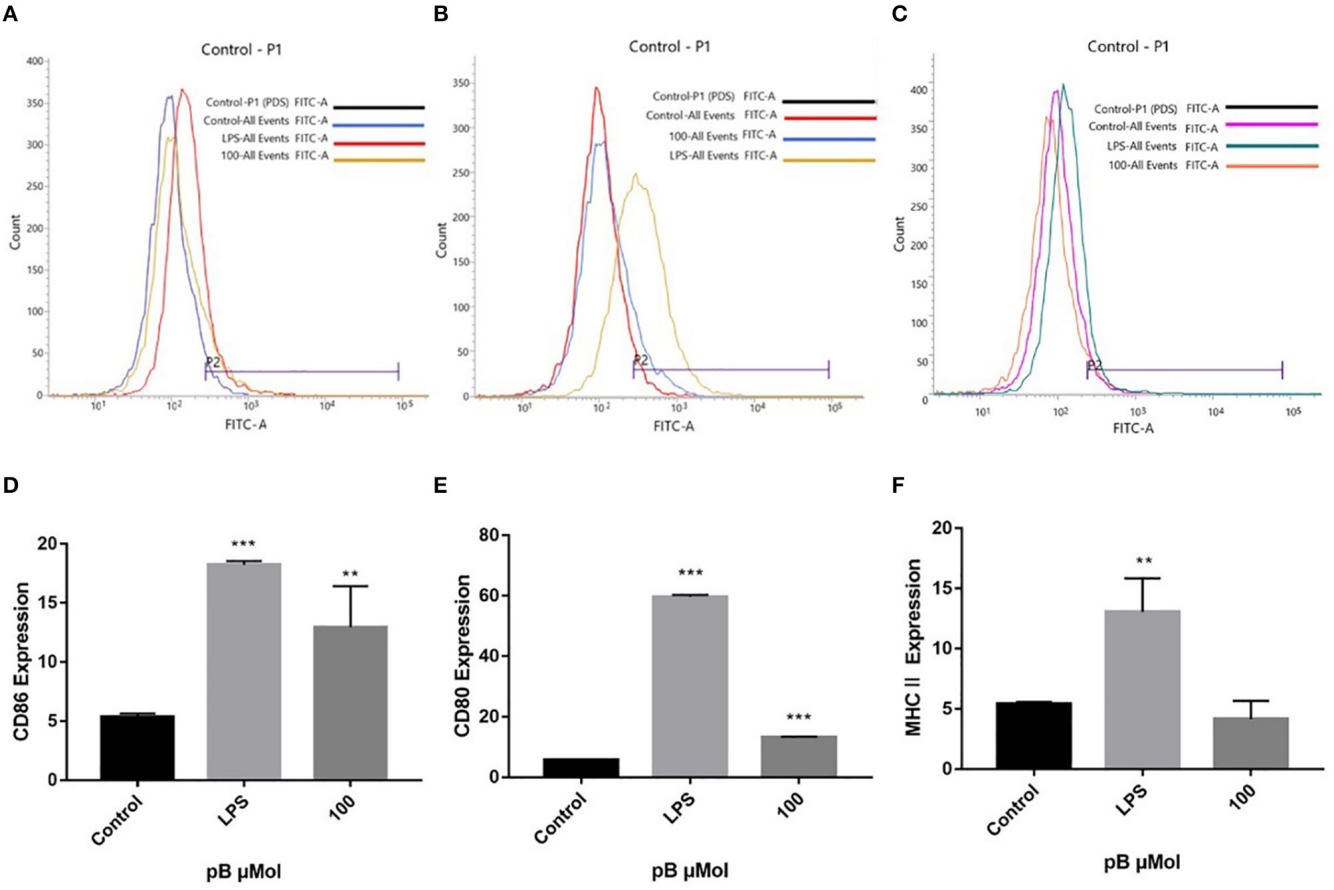
Flow cytometric analysis of CD86, CD80, and MHC II expressions on the surface of RAW264.7 cells. **(A,D)** represent the results of CD86 expression. **(B,E)** represent the results of CD80 expression. **(C,F)** represent the results of MHC II expression. Data presented are the mean ± SD, *n* = 3. Compared with the control, ^***^p < 0.001 and ^**^p < 0.01.

Macrophages act as antigen-presenting cells that load peptides into the MHC-II molecule, subsequently internalizing and processing the antigen, thereby allowing interaction with specific T cell receptors (TCR). However, T cell activation requires that costimulatory molecules CD80 and CD86 bind to CD 28 or CD 152 on the surface of the T cell to complete the immune synapse (35). To explore the effects of pB on the presentation of antigens, we examined the expression of CD86, CD80, and MHC II on the cell surface. In Figure 8, compared with the control group, the expressions of CD86, CD80, and MHC II were significantly increased in the LPS positive group. After pB stimulation, the peaks of CD86 and CD80 shifted significantly to the right, which was significantly different from the blank group (*p* < 0.001). Unfortunately, the peak value of MHC after PB stimulation did not shift to the right, and there was no significant difference compared with the control group. This suggested that pB could upregulate the expression of CD86 and CD80, thus enhancing the antigen-presenting effects of cells.

## Discussion

Immune regulation refers to the interaction between immune cells and immune molecules, and between the immune system and other systems of the body in the process of the immune response, thus forming a mutual coordination and restriction network, sensing the immune response of the body, and implementing regulation to maintain the internal environment stability of the body. NF-κB and MAPKs-signaling pathways are two important pathways in immune signaling. NF-κB is sequestered by IκBα within the cytoplasm of unstimulated cells. After external stimulation, cells activate the IκB kinase (IKK) complex, which then phosphorylates IκBα and leads to the degradation and proteasome of the latter. Then, NF-κB dimer (mainly P50/P65) migrated to the nucleus to induce the expression of target genes and play an immunomodulatory role (36–38). The MAPKs-signaling pathway can also be activated by LPS, including ERK-1/2, P38, and JNK (39). These kinases are jointly activated to promote cell proliferation, migration and invasion, angiogenesis, metastasis, apoptosis, and other functions that are crucial to the development and activation of macrophages and T cells (40, 41). They are closely related but relatively independent and are activated in the form of phosphorylation, thereby jointly playing immunomodulatory roles.

The association between many herbal products and various aspects of immunity has been demonstrated, and the immune-related therapeutic potential of herbal products should not be underestimated. Black pepper (*Piper nigrum*) and cardamom (*Elettaria cardamomum*) have been shown to possess potent immunomodulatory effects (42). *Nigella sativa* and thymoquinone also play an important role in immune regulation (43). Throughout history, people have used extracts of various herbs and plants to prevent disease and boost immunity. Compounds like polysaccharides (44), alkaloids (45), polyphenols (46), flavonoids (47), and other compounds have certain effects on immune regulation. *O. majorana* is an important aromatic plant that is used against cooling, diabetes, allergies, fever, flu, and hypertension. As the ethnomedicinal in Turkey, it is used to treat respiratory tract diseases and as a sedative and a diaphoretic. (6). At present, there are few studies on the immunomodulation of *O. majorana*. In this study, four compounds were isolated from the 40% ethanol extract of *O. majorana*, including 1H-indole-2-carboxylic acid (**1**), (+)-lariciresinol (**2**), (+)-isolariciresinol (**3**), procumboside B (**4**pB). The immune effects of the isolated compounds were screened, and compound **1** had weak immune activity. Compounds **2** and **3** had certain immune activity, which was consistent with previous studies (48, 49). Compound **4** (pB) had good immune activity on RAW264.7 cells and has not been reported. So, the immunomodulatory mechanism of pB has been investigated, and the results showed that pB significantly promoted the secretion of NO, IL-6, and TNF-α, and also increased the expression of the corresponding mRNA. Western blot results showed that pB played an immunomodulatory role by activating NF-κB and MAPKs-signaling pathways. In addition, pB could also promote the production of ROS, and the production of ROS was closely related to the activation of the NF-κB-signaling pathway (19). Thus, we speculated that the activation of the NF-κB-signaling pathway might be related to the production of ROS. We also detected the expression of CD86, CD80, and MHCII on the cell surface by flow cytometry, and the results showed that pB could promote the expression of CD80 and CD86 on the cell surface but did not affect the expression of MHC II. In summary, our results provided further insight into how pB can play a direct role in the regulation of primary macrophage activation early in inflammation.

Procumboside B belongs to the type phenylpropanoid, which has a variety of pharmacological effects, including antitumor, anti-oxidation, antibacterial, antiviral, anti-inflammatory, analgesic, hypotensive, enzyme inhibitory, and immunosuppressive activities (50). The phenylpropanoids in lilacs regulated adipogenesis (51). The anti-inflammatory effect of phenylpropanoids from *Dendropanax dentigerous* was exerted by inhibiting NF-κB, AKT, and JNK signaling pathways in TNF-α-induced MH7A cells (52). Bingyou Yang found that the phenylpropanoids from the fruits of *Nicandra physaloides* showed anti-inflammatory activity (53). However, there are few studies on the immune activity of phenylpropanoid compounds. Our results proved that the phenylpropanoid compound has immune regulation effects.

As the future of nutrition science continues to evolve with rapidly advancing technology and research, the current research team expects the further incorporation of beneficial herbs to be included in general nutrition guidance (54). Changes in nutritional status have a wide range of effects on the body, which can influence organ size, hormone, and cytokine levels and immune cell populations and function (55). Nutrient deficiencies from the inadequate intake of healthful foods can contribute to a weakened immune system and a greater susceptibility to infection. As part of a healthy diet, many plants provide powerful sources of nutrients through antioxidants, polyphenols, polysaccharides, other biologically active compounds, and needed vitamins and minerals (56). At present, people with contemporary dietary patterns may still lack the essential nutrients needed for optimal health, and herbs and spices can provide substantial benefits for overall nutrition due to their high concentration of phytonutrients and other biologically active compounds (57). The Food and Drug Administration (FDA) has recognized herbs and spices as generally recognized as safe (GRAS) for human consumption. Including herbs and spices in a balanced and diverse diet is one of the highlights of nutritious eating that supports health and immunity (56). *O. majorana*, often used as a spice and seasoning in cooking, can help digestion, relieve gas, stop spasms in Egypt, and is essential to our diet. Our results demonstrated the immunomodulatory effects of *O. majorana*. Therefore, the dosage and performance of *O. majorana* can be further investigated, and it can be made into a nutritional supplement additive with immunomodulatory effects and applied to our food and pharmaceutical industry.

## Conclusion

In this study, four compounds in the 40% ethanol fraction of *O. majorana* were isolated and identified, including 1H-indole-2-carboxylic acid (**1**), (+)-laricresol (**2**), (+)-isolaricresol (**3**), and procumboside B (**4**, pB). Immune activities showed that pB had strong immune activity. The mechanism might relate to NF-κB and MAPKs-signaling pathways in RAW264.7 cells, and could also upregulate the expression of CD86 and CD80 to enhance the antigen presentation of macrophages. These results provided the potential of *O. majorana* to be used as a nutritional supplement with immunomodulatory effects.

## Data Availability Statement

The original contributions presented in the study are included in the article/Supplementary Material, further inquiries can be directed to the corresponding author/s.

## Author Contributions

SW and LZ performed the experiments, wrote, and prepared the original draft. QT and MW analyzed and summarized the data. WK and ZL critically reviewed the manuscript. FA contributed to the data acquisition. GW, LL, and ZL supervised project administration. WK provided resources, funding, and reviewed the manuscript. All the authors contributed to the article and approved the submitted version.

## Funding

This work was funded by Research on Precision Nutrition and Health Food, Department of Science and Technology of Henan Province (CXJD2021006).

## Conflict of Interest

The authors declare that the research was conducted in the absence of any commercial or financial relationships that could be construed as a potential conflict of interest.

## Publisher's Note

All claims expressed in this article are solely those of the authors and do not necessarily represent those of their affiliated organizations, or those of the publisher, the editors and the reviewers. Any product that may be evaluated in this article, or claim that may be made by its manufacturer, is not guaranteed or endorsed by the publisher.
