# Supplementary figures and images for: Origanum majorana L.: A Nutritional Supplement With Immunomodulatory Effects
Source: Front Nutr. 2021 Sep 22;8:748031. doi: 10.3389/fnut.2021.748031 (PMC8493290; doi:10.3389/fnut.2021.748031)

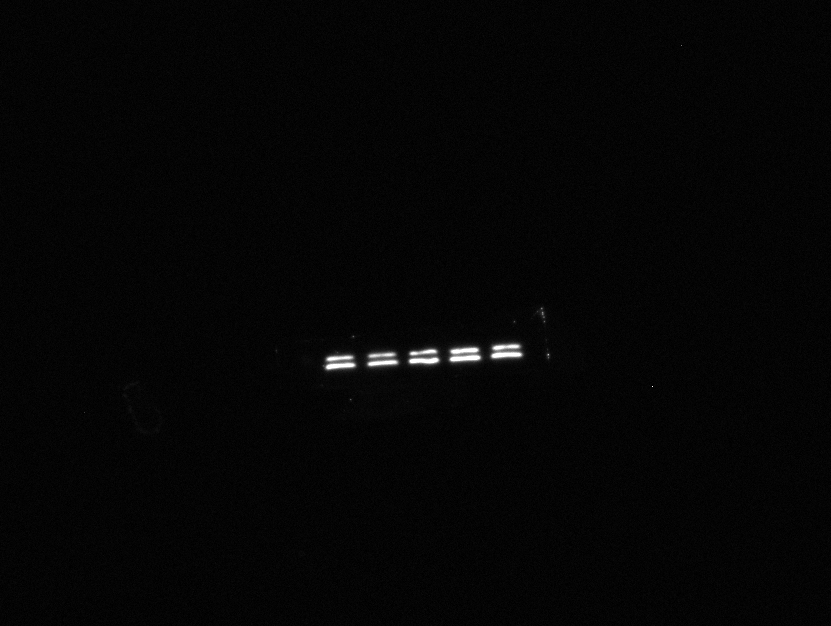

Supplement: Supplementary file 1 [file Data_Sheet_1.ZIP › Raw Data for Origanum majorana L/FIGURE 5 and 6/ERK/contrast_1.png]

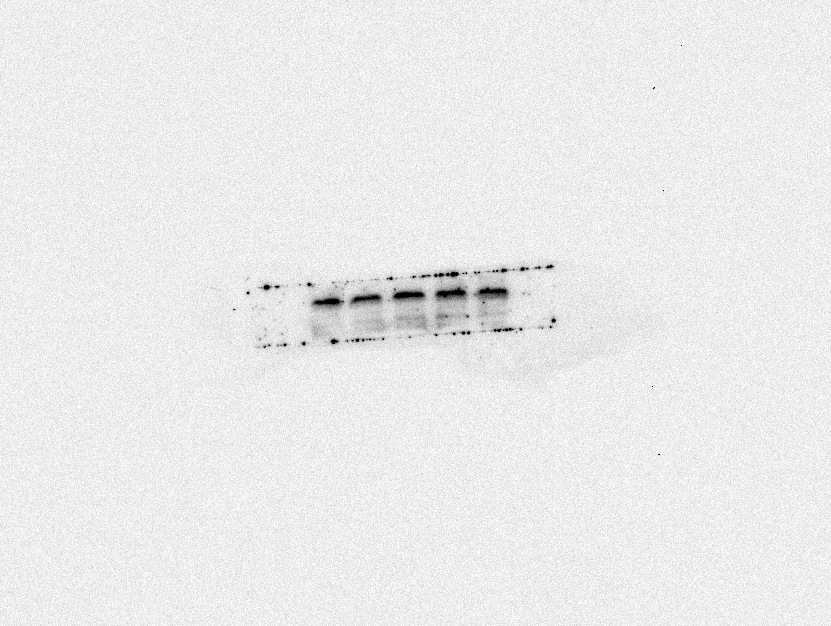

Supplement: Supplementary file 1 [file Data_Sheet_1.ZIP › Raw Data for Origanum majorana L/FIGURE 5 and 6/Ia╩Ba┴/contrast_1.png]

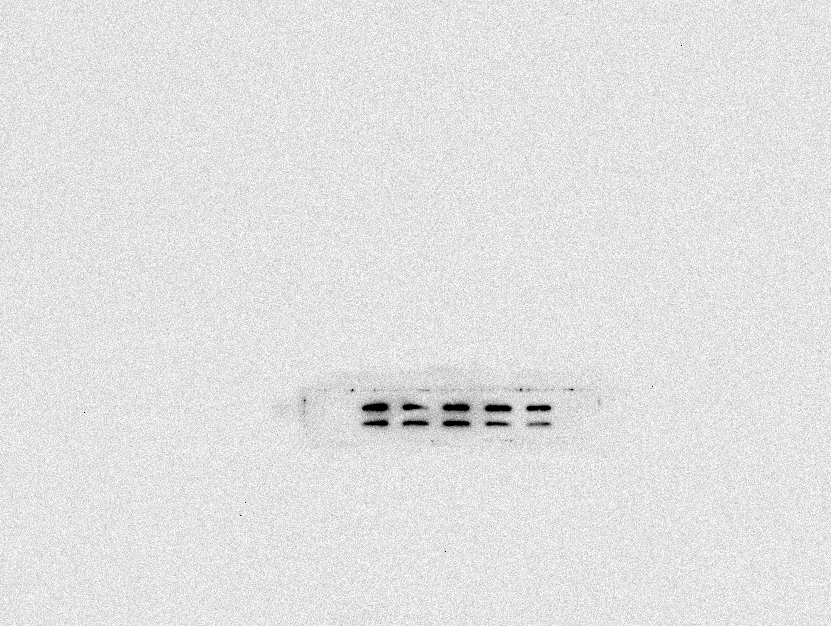

Supplement: Supplementary file 1 [file Data_Sheet_1.ZIP › Raw Data for Origanum majorana L/FIGURE 5 and 6/JNK/contrast_2.png]

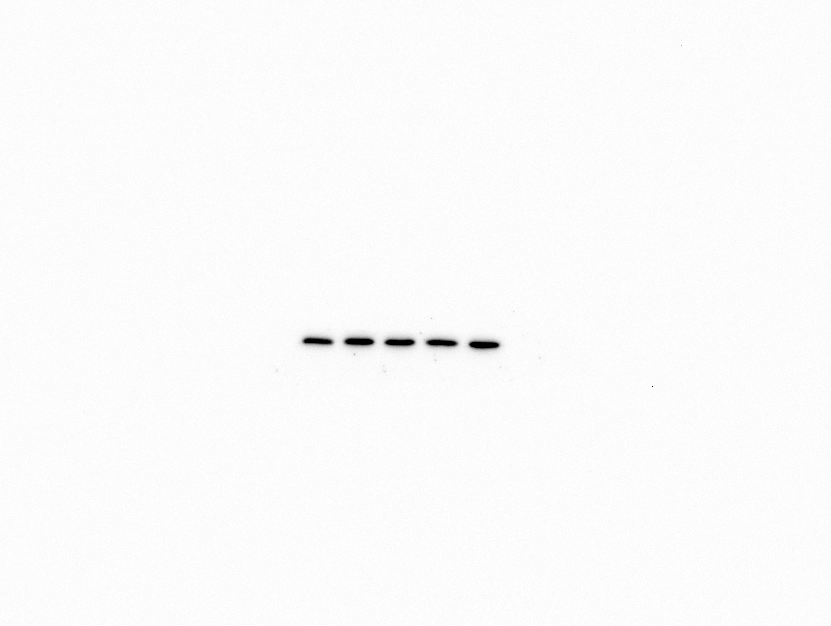

Supplement: Supplementary file 1 [file Data_Sheet_1.ZIP › Raw Data for Origanum majorana L/FIGURE 5 and 6/P38/contrast_1.png]

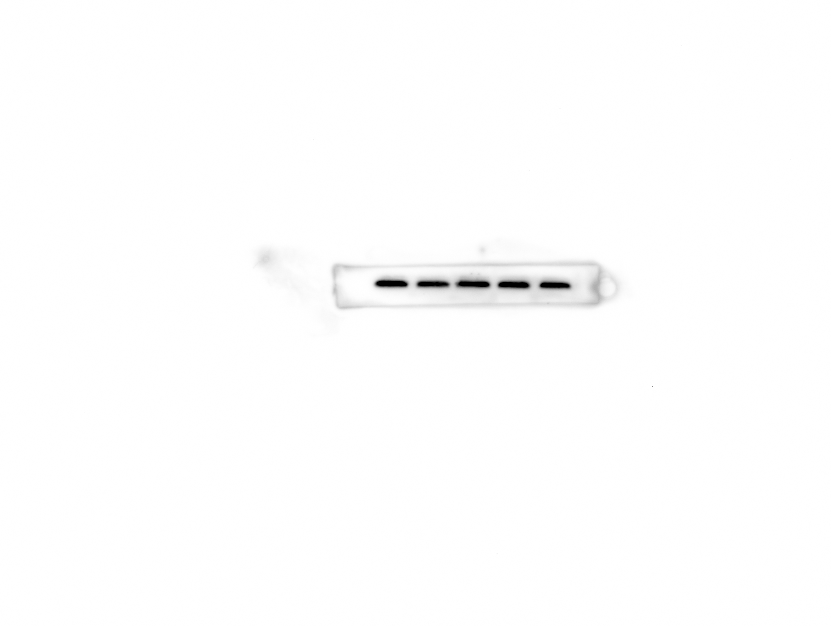

Supplement: Supplementary file 1 [file Data_Sheet_1.ZIP › Raw Data for Origanum majorana L/FIGURE 5 and 6/P65/contrast_1.png]

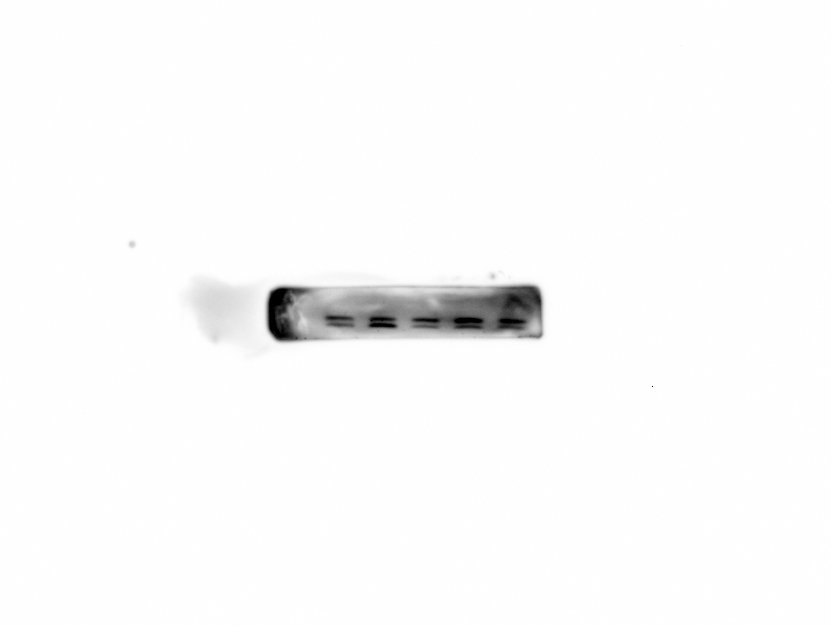

Supplement: Supplementary file 1 [file Data_Sheet_1.ZIP › Raw Data for Origanum majorana L/FIGURE 5 and 6/p-ERK/contrast_1.png]

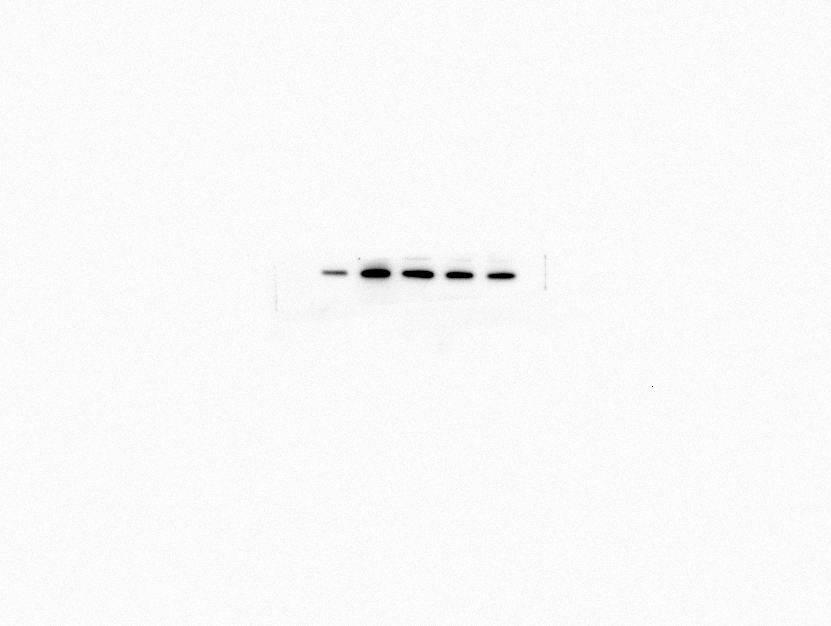

Supplement: Supplementary file 1 [file Data_Sheet_1.ZIP › Raw Data for Origanum majorana L/FIGURE 5 and 6/p-Ia╩Ba┴/contrast_1.png]

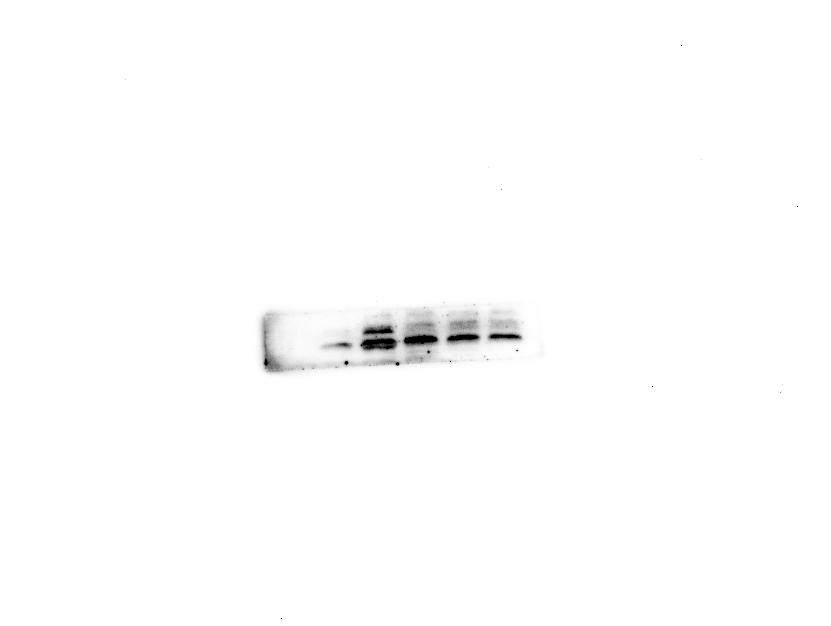

Supplement: Supplementary file 1 [file Data_Sheet_1.ZIP › Raw Data for Origanum majorana L/FIGURE 5 and 6/p-JNK/contrast_1.png]

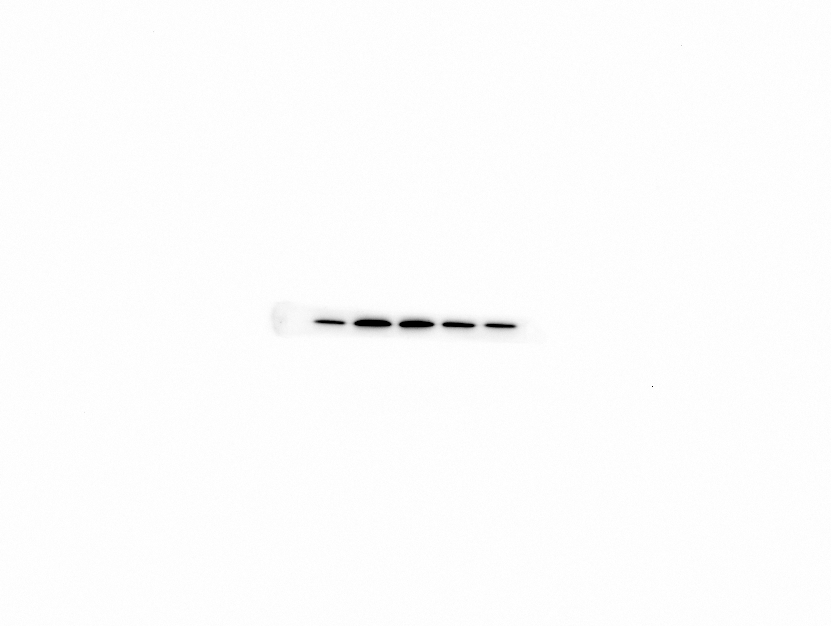

Supplement: Supplementary file 1 [file Data_Sheet_1.ZIP › Raw Data for Origanum majorana L/FIGURE 5 and 6/p-P38/contrast_1.png]

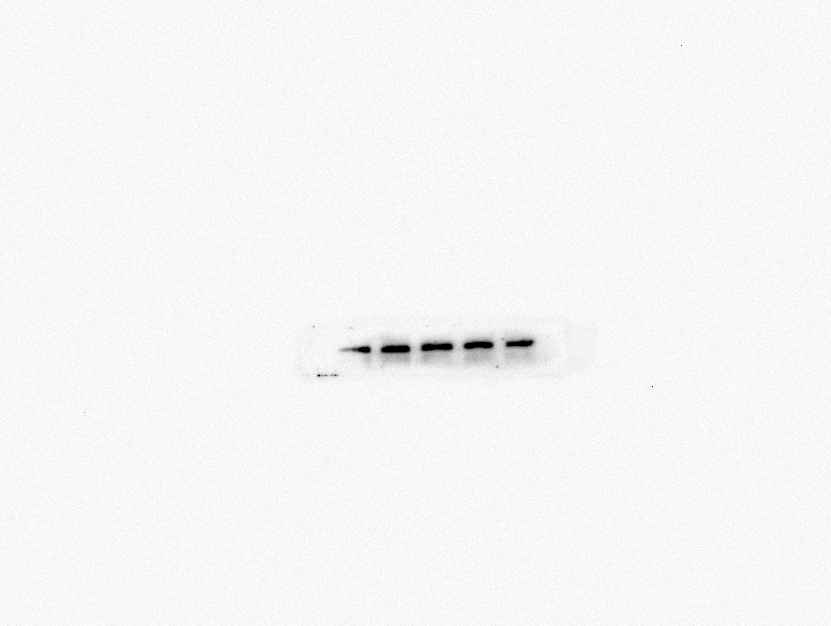

Supplement: Supplementary file 1 [file Data_Sheet_1.ZIP › Raw Data for Origanum majorana L/FIGURE 5 and 6/p-P65/contrast_1.png]

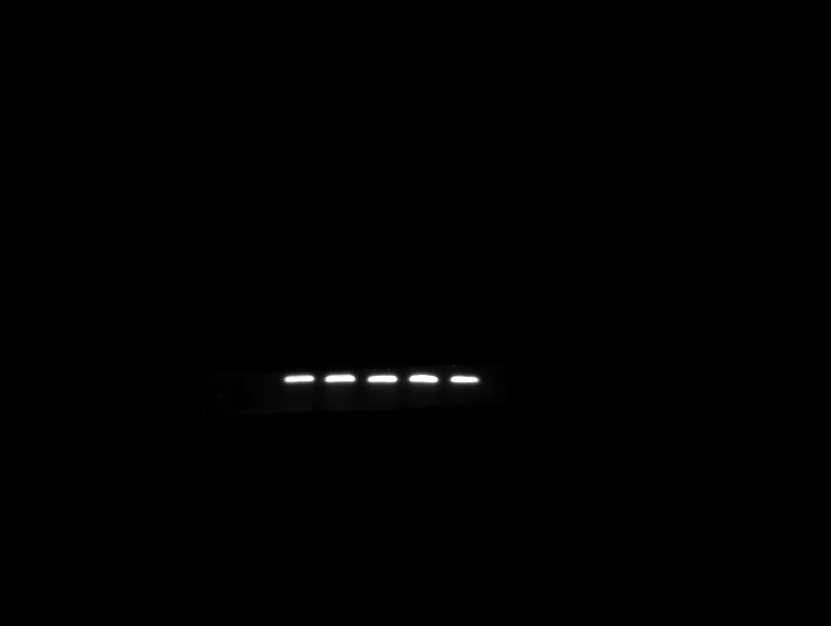

Supplement: Supplementary file 1 [file Data_Sheet_1.ZIP › Raw Data for Origanum majorana L/FIGURE 5 and 6/a┬-actin/contrast_1.png]

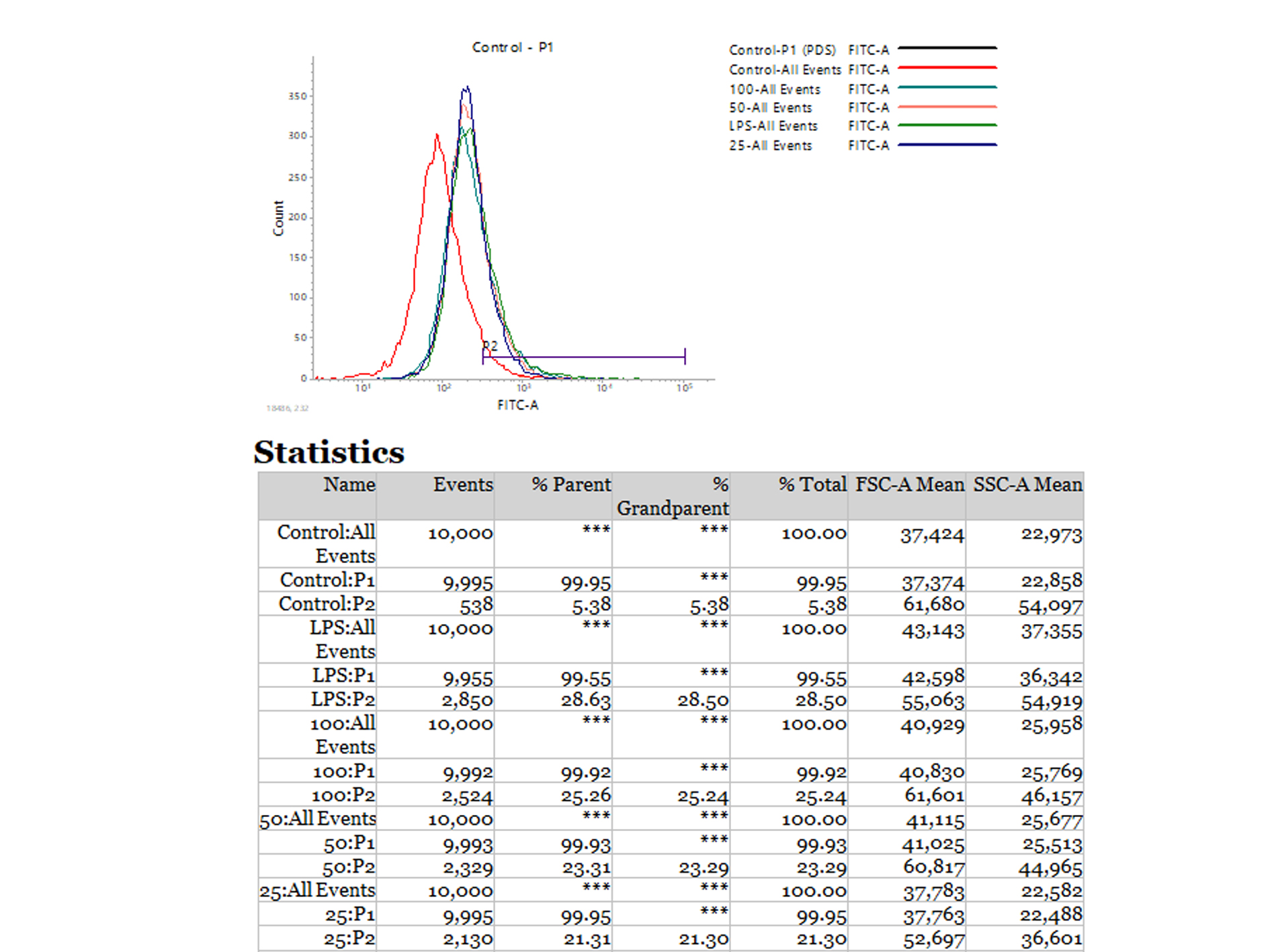

Supplement: Supplementary file 1 [file Data_Sheet_1.ZIP › Raw Data for Origanum majorana L/FIGURE 7/ROS.jpg]

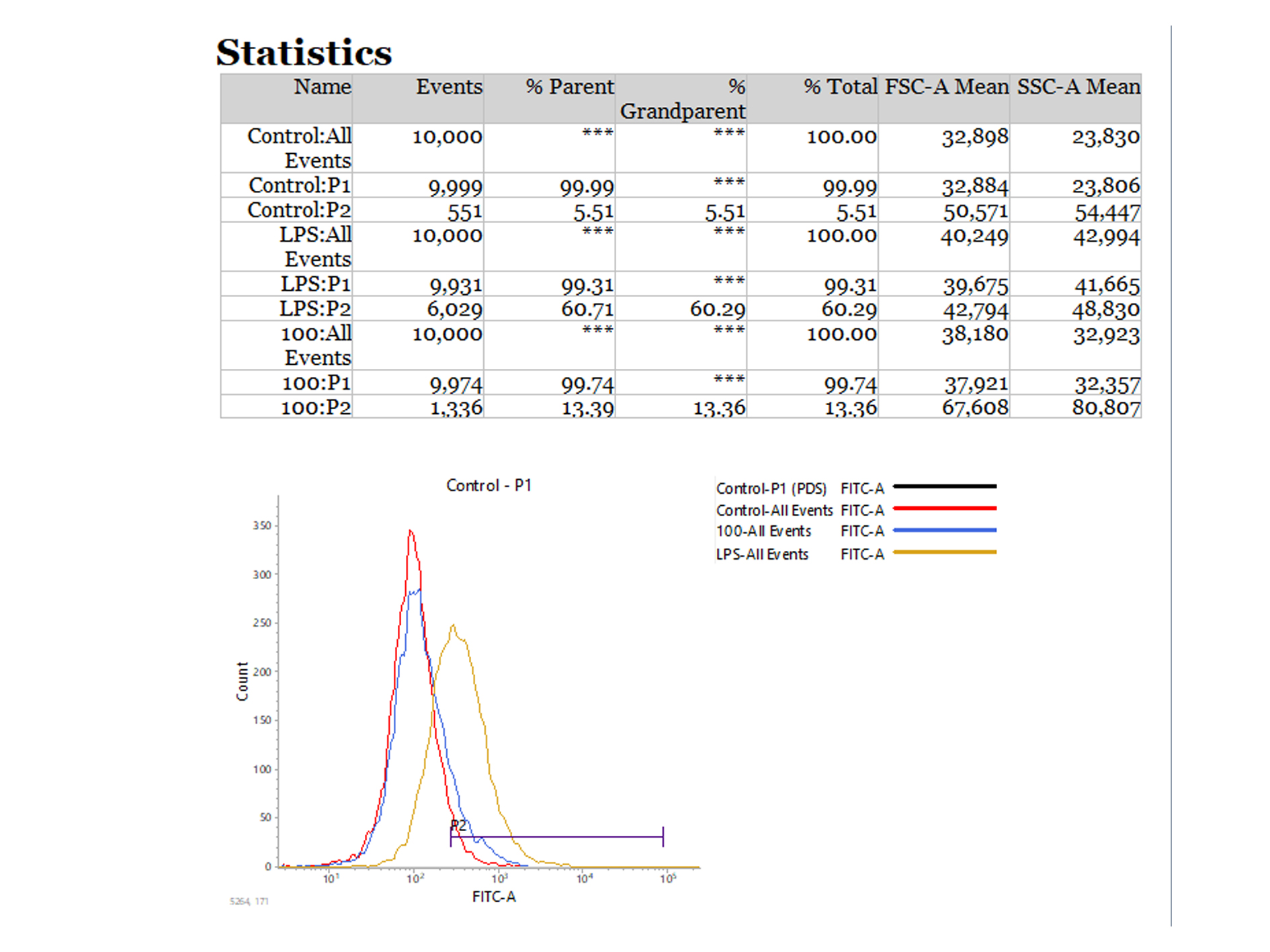

Supplement: Supplementary file 1 [file Data_Sheet_1.ZIP › Raw Data for Origanum majorana L/FIGURE 8/CD80/CD80.jpg]

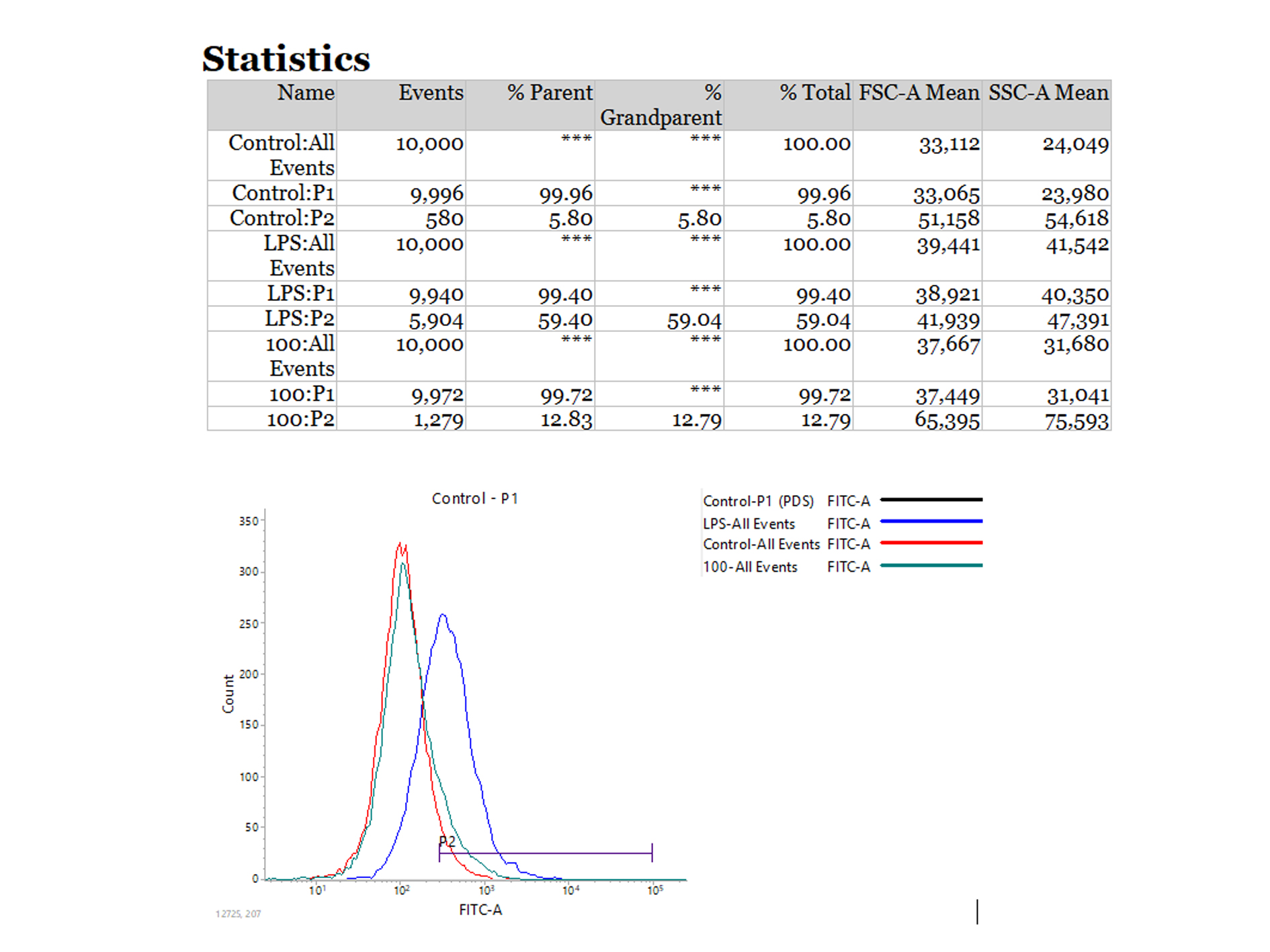

Supplement: Supplementary file 1 [file Data_Sheet_1.ZIP › Raw Data for Origanum majorana L/FIGURE 8/CD86/CD86.jpg]

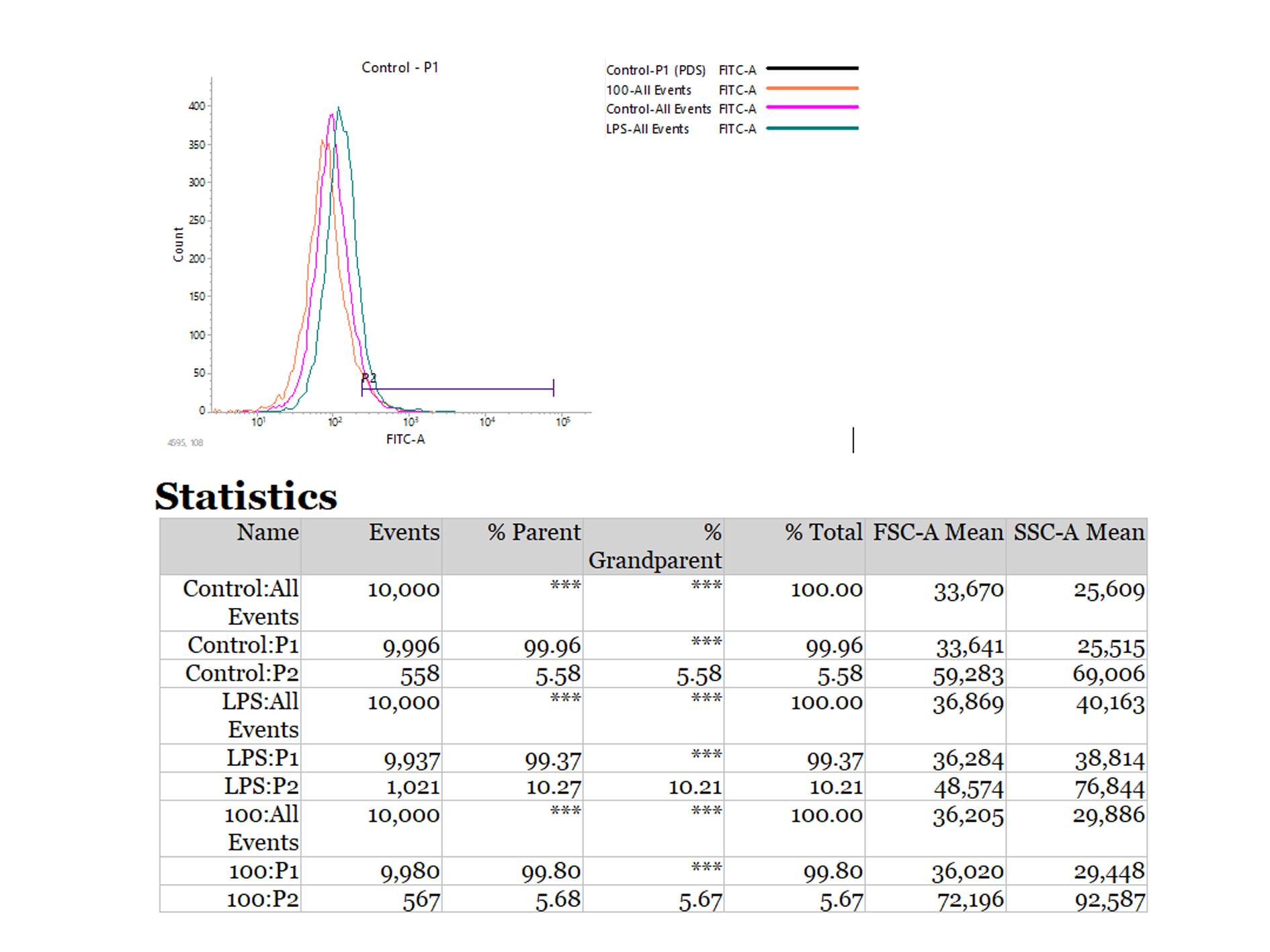

Supplement: Supplementary file 1 [file Data_Sheet_1.ZIP › Raw Data for Origanum majorana L/FIGURE 8/MHC ó≥/MHC-ó≥.jpg]
